# Supplementary material for: Phage Therapy against Staphylococcus aureus: Selection and Optimization of Production Protocols of Novel Broad-Spectrum Silviavirus Phages
Source: Pharmaceutics. 2022 Sep 6;14(9):1885. doi: 10.3390/pharmaceutics14091885 (PMC9503876; doi:10.3390/pharmaceutics14091885)
Supplement: Supplementary file 1 [file pharmaceutics-14-01885-s001.zip › pharmaceutics-1839029-Supplementary Figure S1.pdf]

A.

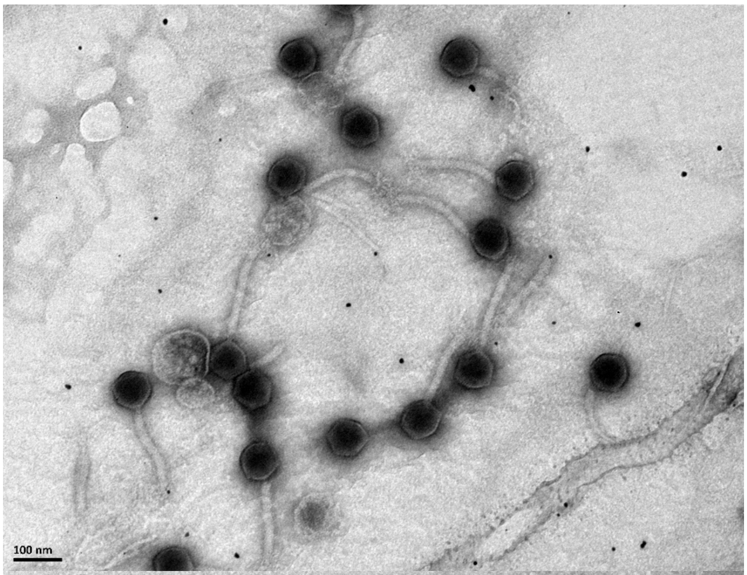

B.

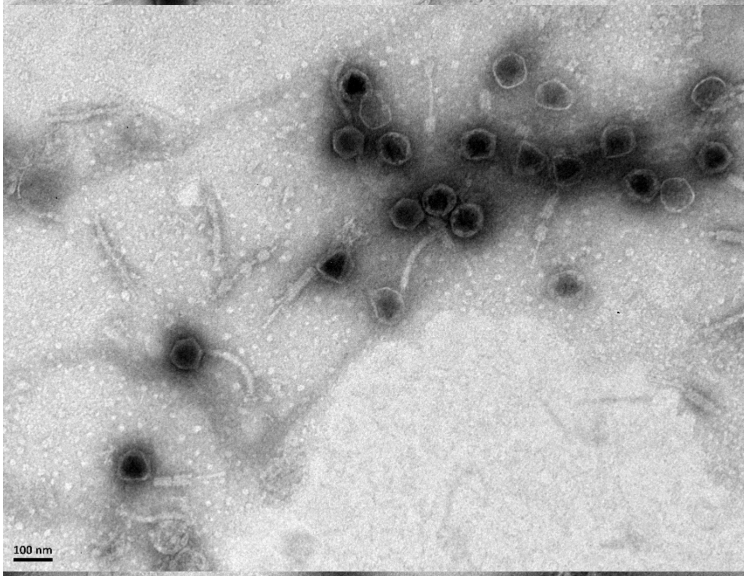

C.

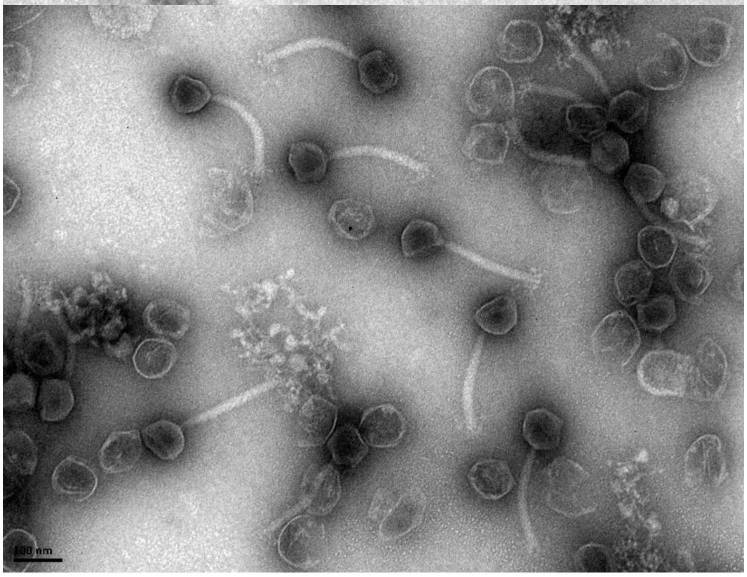

Figure S1._Morphology in TEM of *Silviavirus* anti-*Staphylococcus aureus* phages. (A) V1SA19; (B) V1SA20; (C) V1SA22.
